# Diet fuelling inflammatory bowel diseases: preclinical and clinical concepts

**DOI:** 10.1136/gutjnl-2021-326575

**Published:** 2022-09-16

**Authors:** Timon E Adolph, Jingwan Zhang

**Affiliations:** 1 Department of Medicine I, Gastroenterology, Hepatology & Metabolism, Medical University Innsbruck, Innsbruck, Austria; 2 Department of Medicine & Therapeutics, The Chinese University of Hong Kong, Hong Kong, Hong Kong

**Keywords:** inflammatory bowel disease, diet, gut inflammation, dietary factors, intestinal microbiology

## Abstract

The diet and gut microbiota have been extensively interrogated as a fuel for gut inflammation in inflammatory bowel diseases (IBDs) in the last few years. Here, we review how specific nutrients, typically enriched in a Western diet, instigate or deteriorate experimental gut inflammation in a genetically susceptible host and we discuss microbiota-dependent and independent mechanisms. We depict the study landscape of nutritional trials in paediatric and adult IBD and delineate common grounds for dietary advice. Conclusively, the diet reflects a critical rheostat of microbial dysbiosis and gut inflammation in IBD. Dietary restriction by exclusive enteral nutrition, with or without a specific exclusion diet, is effectively treating paediatric Crohn’s disease, while adult IBD trials are less conclusive. Insights into molecular mechanisms of nutritional therapy will change the perception of IBD and will allow us to enter the era of precision nutrition. To achieve this, we discuss the need for carefully designed nutritional trials with scientific rigour comparable to medical trials, which also requires action from stake holders. Establishing evidence-based dietary therapy for IBD does not only hold promise to avoid long-term immunosuppression, but to provide a widely accessible therapy at low cost. Identification of dietary culprits disturbing gut health also bears the potential to prevent IBD and allows informed decision making in food politics.

**WHAT IS ALREADY KNOWN ON THIS TOPIC**

- The diet and host immune responses determine gut microbial composition and function.
- Excessive intake of specific macronutrients enriched in a Western diet promotes experimental gut inflammation by perturbation of host–microbe commensalism.
- Dysbiosis in inflammatory bowel diseases (IBDs) is fuelling experimental gut inflammation.
- Clinical trials indicate that the diet affects gut inflammation in patients with IBD.

**WHAT THIS STUDY ADDS**

- This review summarises recent experimental and clinical advances on the role of the diet in IBD

**HOW THIS STUDY MIGHT AFFECT RESEARCH, PRACTICE OR POLICY**

- Patient-tailored dietary advice will be a cornerstone to prevent and treat IBD in the future.

## Introduction

Inflammatory bowel diseases (IBDs) encompass a spectrum of chronic inflammatory disorders in and beyond the gut, typically referred to as Crohn’s disease (CD) or ulcerative colitis (UC).^1^ Today, these diseases emerged across the globe, which was paralleled by Westernisation of lifestyle and particularly the diet, while a specific environmental factor that would trigger or affect the course of IBD in a genetically susceptible individual remains obscure.^2^ The prevalence of IBD is expected to rise to 1% in developed and newly industrialised countries, indicating the need for a better understanding of these relapsing diseases.^3–5^ In the last decade, clinical studies established the efficacy and safety of immunosuppressive therapy (with biologicals and small molecules),^6^ while at the same time, the mechanistic basis of experimental diet-induced gut inflammation was increasingly delineated.^7–9^ A vast body of recent evidence indicates that Western dietary constituents and excess of macronutrients fuel experimental gut inflammation, by directly impacting gut mucosal immune responses or by alterations of the gut microbiota.^9 10^ Consequently, compositional and functional alterations of the gut microbiota, collectively termed dysbiosis, have been identified as a fuel for gut inflammation in experimental models and possibly IBD.^11 12^ It is notable that these disease concepts have been similarly described for obesity and related disorders.^13–15^ In line with this, prospective epidemiological studies indicated that obesity emerges as an independent risk factor for CD.^16^ Preclinical and translational studies indicated that energy metabolism controls gut immune responses and that excessive intake of carbohydrates and long-chain fatty acids deteriorate or instigate gut inflammation in several mouse models.^9^ In human IBD, early surgical studies from the 1990s indicated that luminal factors (potentially nutrients, microbes or related metabolites) are sufficient to evoke gut inflammation,^17 18^ which led to the nowadays established therapeutic concept of ileostomy. At the same time, early nutritional trials indicated that dietary therapy with exclusive enteral nutrition (EEN) (ie, enteral feeding with formula diets) effectively induces remission in paediatric and possibly adult patients with CD.^19^ Collectively, these studies led to the appreciation of the metabolic nature of IBD.^9^ In comparison to rapidly evolving medical therapies during the last two decades, nutritional trials failed to establish unequivocal evidence for dietary advice (beyond EEN) that would ameliorate the course of IBD in adults. However, evidence from recent experimental, epidemiological and nutritional trials supported a critical role for the diet as a fuel for gut inflammation in IBD.^10 20^ In this review, we conceptually summarise evidence for the diet as a critical rheostat of experimental gut inflammation. Moreover, we depict the study landscape of nutritional trials in IBD and delineate common ground for a dietary approach. Finally, we discuss the need for carefully designed nutritional trials that can compare with medical trials, which makes it necessary to revisit our nutritional approach today.

## Excessive intake of specific nutrients or additives in a Western diet drives gut inflammation in preclinical models

Western dietary habits are characterised by increased intake of fat and simple carbohydrates, and reduced intake of plant-derived complex carbohydrates (ie, fibre). Recent experimental evidence indicated that specific macronutrients in a Western diet deteriorate experimental gut inflammation that is induced by genetic or chemical means (box 1). Prime evidence for the concept of diet-induced immune perturbation in a genetically susceptible host was provided by Devkota and colleagues, demonstrating that milk fat exposure deteriorates colitis in mice that lack the anti-inflammatory cytokine interleukin 10 (*IL10^−/−^*), by the bloom of the gut pathobiont *Bilophila wadsworthia*.^21^ Subsequent studies indicated that a Western style diet impairs epithelial barrier function in mice and susceptibility to chemically induced (toxic) colitis.^22–24^ In line with this concept, a glucose-enriched diet deteriorated colitis in *IL10^−/−^* mice and toxic colitis,^25^ which was similarly noted for fructose and sucrose enrichment.^26–29^ Not only macronutrients, but also food additives typically enriched in a Western diet (and a related lifestyle) promote susceptibility to colitis. For example, supplementation of food colourants Red40 (E129) and Yellow 6 (E110) drive colitis in mouse models in which IL-23 expression mediated gut inflammation.^30^ These colourants are contained in soft drinks, candy, sauces and dairy products. A second example for a critical role of food additives are emulsifiers which are used to stabilise food in a single phase (eg, in oil-in-water solutions such as mayonnaise or margarine). Chassaing and colleagues demonstrated that carboxymethylcellulose (E466) and polysorbate-80 (E433) promote susceptibility to colitis in *IL10^−/−^* mice.^31^ Moreover, the additive maltodextrin (E1400, a thickener used in instant pudding, gelatins, sauces and dressings) deteriorates experimental colitis in mice.^32^ Notably, in some of these experimental approaches, the gut microbiota (or its metabolites) mediated the inflammatory effects of the diet (beyond association),^21 25 31^ as also demonstrated for fungi in a mouse model of gut injury.^33^ Indeed, gut microbial dysbiosis of bacteria, fungi and viruses (bacteriophages) is a hallmark of IBD (see the next section), which exerts pro-inflammatory functions when transplanted into genetically susceptible *IL10^−/−^* mice.^34^ In turn, an EEN formula enriched with specific prebiotics ameliorates experimental (adoptive T-cell transfer) colitis, which could be partly explained by restoration of bacterial communities.^35^

Box 1 **Nutrients fuelling gut inflammation**

A Western diet is enriched with simple carbohydrates (eg, fructose, sucrose) and fat (eg, long-chain fatty acids such as arachidonic acid), while being largely devoid of fibre.^148^ Moreover, a Western diet is enriched with emulsifiers and food colourants contained in processed food. Experimental evidence indicates that diets enriched with carbohydrates or fat deteriorate gut inflammation, similar to emulsifiers and food colourants. These dietary constituents either directly trigger mucosal immune responses, for example, in susceptible epithelial cells, or indirectly modulate mucosal immune responses during gut inflammation by affecting the microbiota. For example, dietary polyunsaturated fatty acids are oxidised at the endoplasmic reticulum in small intestinal epithelial cells, which triggers toll-like receptor 2 activation and an acute inflammatory response in the gut, which is restricted by cellular hubs known to be compromised in CD.^36 38^ Likewise, polyunsaturated fatty acids directly trigger the expression of cytokines in susceptible Crohn’s epithelium^38^ and fibroblasts.^149^ In turn, dietary restriction with an elemental diet (which may reduce an excess of Western dietary constituents) induces remission in paediatric (and possibly also adult) patients with CD.^41 129 150^ These experimental and clinical studies indicate that the diet serves as a critical fuel for gut inflammation in IBD.

Western dietary constituents also directly impact gut mucosal immune responses. For example, we recently demonstrated that long-chain polyunsaturated fatty acids (PUFAs), contained in red and white meat, eggs and cooking oils, trigger an inflammatory response from gut epithelial cells, which is restricted by Glutathione peroxidase 4 (GPX4).^36^ GPX4 is an evolutionary conserved anti-oxidative enzyme with activity towards PUFAs that protects against lipid peroxidation and related sequelae.^37^ Mice that display reduced intestinal epithelial GPX4 activity, which models the epithelium from patients with CD, develop enteritis resembling aspects of human CD when exposed to PUFAs in a Western diet.^36^ Enteritis is mediated by toll-like receptor 2 sensing of lipid peroxides (induced by ω−3 and ω−6 PUFAs), which instigates endoplasmic reticulum stress and expression of the IL-8 homologue CXCL1 in this model.^38^ Importantly, PUFA exposure evoked an inflammatory response from CD epithelium with impaired GPX4 activity and estimated PUFA intake correlated with a poor course of CD.^38^

**Figure 1:**
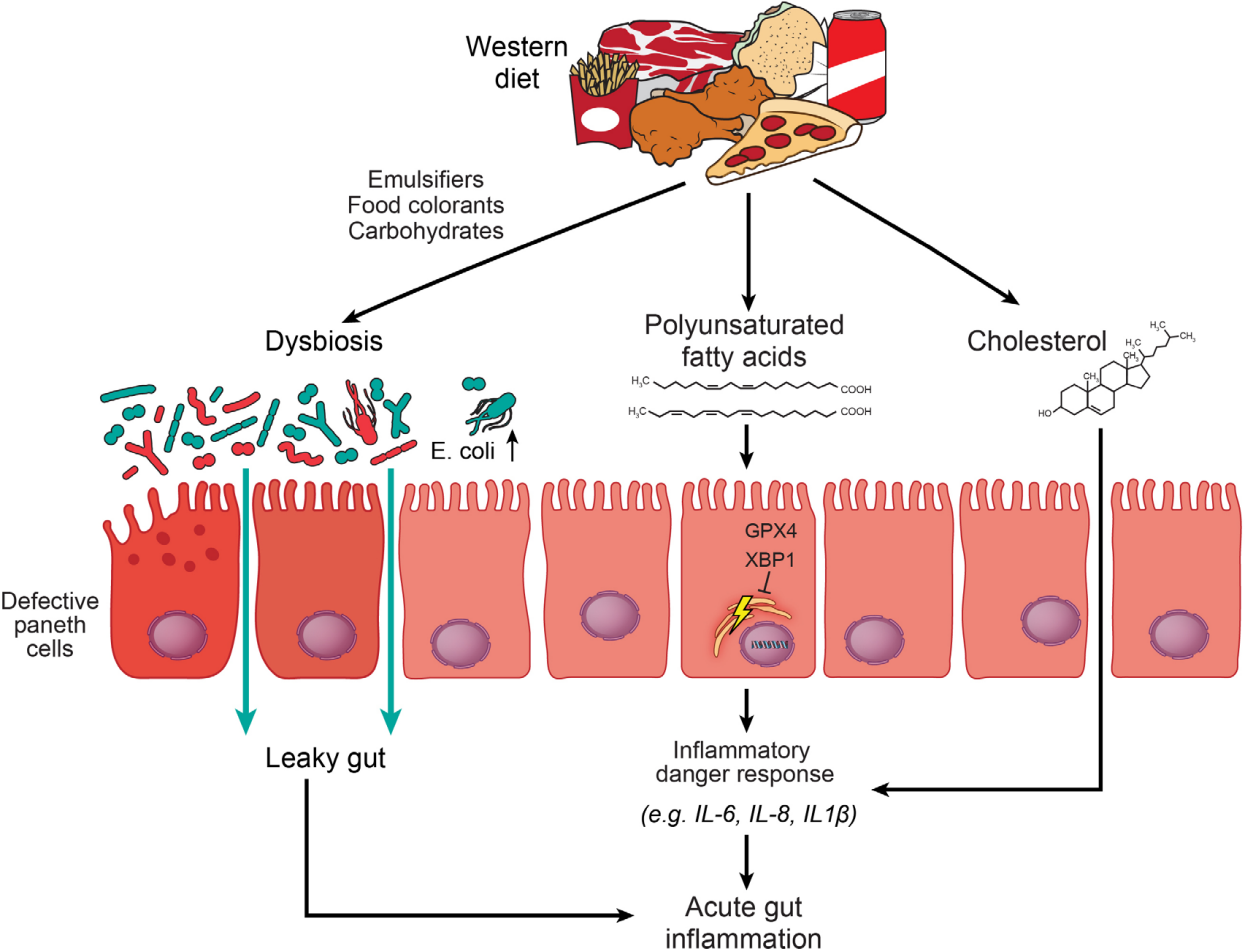
The Western diet impairs epithelial immune responses and promotes dysbiosis and inflammation. A Western diet is enriched with simple carbohydrates, fat (eg, saturated and polyunsaturated fatty acids and cholesterol) and food additives (eg, emulsifiers, food colourants, processed carbohydrates). These compounds may directly induce compositional and functional alterations of the gut microbiota, which partly impairs epithelial functions in the gut, that is, perturbs Paneth cells and the gut barrier.^151^ Consequently, a dysbiotic microbiota promotes susceptibility to gut inflammation by perturbation of host–microbe interactions.^21 30 31^ Polyunsaturated fatty acids in a Western diet trigger acute enteritis in mice without evidence for gut microbial dysbiosis, which is rather controlled by epithelial endoplasmic reticulum homeostasis (maintained by X-box-binding protein 1 and Glutathione peroxidase 4).^36 38^ Cholesterol exposure induces an acute inflammatory response involving neutrophils in the gut of mice, possibly by inflammasome sensing.^152^ GPX4, Glutathione peroxidase 4; IL, interleukin; XBP1, X-box-binding protein 1.

Collectively, these studies demonstrated that excessive intake of specific nutrients and additives in a Western diet, such as PUFAs, simple carbohydrates and food colourants, trigger or deteriorate experimental gut inflammation, by exploiting the gut microbiota, or by engaging innate immune receptors and related cellular stress signalling (figure 1). As such, a strength of these approaches is to pin down a specific dietary factor that controls gut inflammation, and to gain mechanistic insights how the diet affects gut health in a genetically susceptible host. A weakness is that the relevance of an experimental approach for human disease often remains unresolved, highlighting the need to go beyond associations, which requires nutritional trials. That this can be rewarding has been demonstrated by recent dietary intervention studies in paediatric and adult CD, which indeed provide evidence for nutritional therapy. For example, EEN with or without a specific exclusion diet (which seeks to correct Western dietary habits) potently induces disease remission in mild-to-moderate CD as outlined below.^39–43^ These studies thus support the concept that the diet is a central rheostat of gut inflammation in IBD. Future studies will expand the growing list of detrimental food constituents that may trigger or deteriorate gut inflammation in preclinical models, and translational efforts should be made to demonstrate a direct role for nutrients during gut inflammation in patients with IBD.

## The diet is a critical determinant of gut microbial composition and function in mice and humans

In IBD, altered microbial signatures (of bacteria, viruses and fungi) have been consistently reported^44–47^ and there is little doubt that industrialisation goes along with gut microbial alterations in humans.^48^ For example, metagenomic studies in IBD indicated a reduction in diversity with lower proportion of *Firmicutes* and increased abundance of *Proteobacteria* and *Bacteroidetes* phylum members in stool, with specific species and related metabolic pathways emerging.^49 50^ Conceptually, gut microbial dysbiosis in human IBD drives gut inflammation in mammals,^34^ and, besides genetic susceptibility, emerges as a key rheostat of gut inflammation.^51^ Dysbiosis can result from a bloom of pathobionts and/or disappearance of beneficial symbionts, which may act on gut barrier functions, perturb immune responses or gut metabolism.^51 52^ However, our current understanding of how gut microbes mechanistically intersect with gut inflammation in IBD is scarce,^53^ which was partly overcome by unbiased multilayered analyses of large IBD cohorts (ie, 1595 metagenomes, 800 paired metatranscriptomes and 201 metaproteomes of the human gut microbiota). One example of a perturbed host–microbe commensalism in IBD involves microbial bile acid metabolism. Dysbiosis has been linked to an increased primary (and reduced secondary) bile acid pool in IBD.^54^ This is notable because bile acids show potent immunomodulatory functions in the gut,^54 55^ and gut microbes in humans specifically modulate TH17 cell immune responses via bile acid metabolites.^56^ Moreover, secondary bile acids allow prediction of remission with biological therapies.^57^ Apart from regulating bacterial communities, diet can also affect bacterial metabolism. For example, a salt-enriched diet has been linked to a particular protein expression panel secreted by bacteria.^58^ Indeed, recent evidence indicates that IBD is associated with a distinct profile of well-characterised and un-characterised bacterial proteins,^59^ and—in case of pathogens—microbes use complex secretion systems to deliver virulence proteins to disrupt cellular functions of the host.^60 61^

The diet is thought to strongly contribute to microbial variation in mice (~50%) and humans ~20%, with strong differences between individuals.^62^ As such, it is conceived that microbial variation could explain some heterogeneity between patients with IBD. In the following section, we describe how dietary constituents affect gut microbial community structures and gut inflammation in mice and humans.

### Carbohydrates

Carbohydrates are generally classified as digestible and non-digestible and are contained in a wide range of food items. Digestible carbohydrates can be enzymatically degraded into simple sugars that are largely absorbed in the small intestine and passed into the bloodstream through the portal vein.^63^ Non-digestible carbohydrates, for example, fibre and resistant starch, are not absorbed in the small intestine but undergo fermentation in the large intestine by resident microorganisms, which provide the host with an energy and carbon source.^64 65^ Experimental approaches demonstrated that excessive intake of simple carbohydrates promoted dysbiosis and gut inflammation (see above). However, the role of excessive simple carbohydrates in the development or course of IBD is poorly explored. In contrast, complex carbohydrates (typically derived from vegetables), and their bacterial metabolites, rather exert a protective effect. For example, bacterial short chain fatty acid (SCFA) generation such as butyrate (through fermentation of complex carbohydrates) allows to maintain gut homeostasis^66^ by protecting intestinal barrier integrity and host immune responses. For example, SCFAs stabilise HIF-1,^67^ a transcription factor coordinating barrier protection^68^ and supplementation of butyrate-producing bacteria, especially *Butyrococcus pullicaecorum*, improved epithelial barrier integrity in CD based on simulations.^69^ Moreover, butyrate also exerts anti-inflammatory effects in the gut mucosa by inhibition of histone deacetylases and activation of G protein-coupled receptors present in gut epithelium and mucosal immune cells.^70 71^ Low fibre intake has been associated with increased IBD risk,^72–74^ and patients with IBD show a decrease in butyrate producing bacterial species, as well as a decreased expression of butyrate transporters.^75–77^ A reduction of butyrate-producing bacteria and the dietary substrate for SCFA generation in patients with IBD may lead to loss of an anti-inflammatory ‘break’ in the gut. In turn, it appears plausible that butyrate supplementation ameliorates the course of IBD, which is currently probed by several clinical trials with butyrate in IBD.

### Fat

Human studies indicated that a high-fat diet increases anaerobic abundance of, for example, *Bacteroides*.^78 79^ Fifteen clinical studies (including six randomised controlled interventional studies and nine observational studies) have shown that total fat or saturated fat suppressed richness and diversity of the gut microbiota.^80^ As such, it is conceived that a high-fat Western diet is a key driver of gut dysbiosis,^81 82^ which may promote gut inflammation as evidenced by studies in humanised mice.^22^ The impact of specific bacterial strains (blooming during Western dietary habits) on gut inflammation in IBD requires further studies.

### Protein

Dietary proteins are derived from plants and animals. Several culture-based studies demonstrated that consumption of whey and pea protein extracts facilitates growth of *Bifidobacterium* and *Lactobacillus*, while whey impairs abundance of *Bacteroides fragilis* and *Clostridium perfringens* in humans.^83–85^ The essential amino acid tryptophan (in dietary protein), which is catabolised by the colonic gut microbiota, controls bacterial communities and the gut immune system (through aryl hydrocarbon receptor signalling).^86^ In contrast to plant-based protein, the abundance of bile-tolerant anaerobes such as *Bacteroides*, *Alistipes* and *Bilophila* increased following consumption of animal-based protein.^87–89^ Animal-based protein enhanced the sensitivity to experimental gut inflammation possibly by expansion of rather inflammatory strains such as *Escherichia*, *Streptococcus* and *Enterococcus*.^90^ In line with this notion, replacement of animal protein with plant protein in a Western diet protected against experimental gut inflammation characterised by an increased *Lactobacillaceae* and *Leuconostraceae* abundance.^91^ In IBD, the role of dietary protein (and related microbial alterations) appears unresolved.

### Food additives

Food additives preserve a food product (in terms of safety, freshness, texture or appearance) or enhance the taste of processed food. Emerging evidence indicates that the consumption of food additives perturbs microbial composition and promotes experimental gut inflammation (also see the above section). For example, artificial sweeteners such as saccharin promote dysbiosis in mice (with increased *Bacteroides* and reduced *Lactobacillus* spp),^92^ which was similarly notable in humans.^93^ The sweetener Splenda deteriorated experimental gut inflammation in SAMP1/YitFc (SAMP) mice, which was accompanied by overgrowth of *Proteobacteria* and *Escherichia coli*.^94^ Likewise, emulsifiers perturb gut microbial community structure and promote susceptibility to gut inflammation, with increased abundance of *Porphyromonadaceae* spp in faeces of P80 fed mice.^95^ Emulsifier also evoked alterations of the gut microbiota in humans.^96^ Moreover, titanium dioxide, usually used as a white powder of different particle sizes (E171) in candies, sweets, pastries and sugar-coated chewing gums, impairs gut permeability and potentially promotes gut inflammation as excellently reviewed recently.^97^ Finally, food colourants Red40 (E129) and Yellow 6 (E110) drive colitis in mouse models with IL-23 expression, which was mediated by metabolism of these colourants in commensals (*Bacteroides ovatus* and *Enterococcus faecalis*).^30^ The role of food additives on the development or course of human IBD is poorly explored. However, it is conceivable that additives contribute to dysbiosis in human IBD which may act as a fuel for gut inflammation.

Collectively, excessive intake of specific food constituents in a Western diet may be a potent trigger of gut dysbiosis in humans (eg, by increased intake of calories derived from fat, digestible carbohydrates, animal protein and food additives), and IBD-associated dysbiosis exerts inflammatory functions in genetically susceptible mice. However, several aspects are poorly resolved in this context. For example, what is the specific impact of blooming pathobionts or loss of symbionts in human IBD and can this be therapeutically exploited? Critical mechanistic insights are probably best exemplified by studies on adherent invasive *E. coli*.^98^ Moreover, current human studies rarely delineate, which genetic susceptibility is required to elicit diet-induced gut inflammation, with or without dysbiosis, in patients at risk for IBD.^99^ And finally, other environmental influences (possibly also in early life) impact gut microbial functions,^49^ such that the diet emerges as one, but not sole rheostat of dysbiosis in IBD. Despite these unresolved issues it was conceived that a specific dietary pattern could be used to reverse microbial perturbation and to ameliorate gut inflammation in IBD, which has been explored by recent dietary intervention trials.

## Dietary interventions in IBD

Experimental, translational and clinical evidence suggest that IBD arises from unresolved perturbation of mucosal immune responses that is determined by genetic variation and the exposome (including the diet and gut microbiota). This concept implies that a variety of cues, rather than a single incident, promotes the development of chronic unresolved gut inflammation, which may explain heterogenous results of key dietary intervention trials (and medical trials alike), which are summarised in table 1.^100^ As such, recent guidelines explicitly state that there is no ‘IBD diet’ that can be generally recommended to induce or maintain remission in patients with IBD.^101^ However, affected individuals suspect a critical role of the diet for their disease.^102^ In line with this, a specific Western dietary pattern (characterised by consumption of grain products, oils, potatoes, processed meat, condiments and sauces, and sugar, cakes and confectionery) was associated with the risk for developing a UC flare during an observational period of 2 years in 427 patients that were in remission at study inclusion.^103^ In turn, EEN (which replaces solid food with a liquid elemental diet) is effective in paediatric (and possibly adult) CD, which is however difficult to adhere (see below). In contrast to the notion that the diet may act as a fuel for gut inflammation in IBD, unequivocal evidence indicates that malnutrition (usually alluding to energy and/or nutrient deficiency consequent to gut inflammation) commonly affects patients with IBD and comes along with increased mortality,^104^ and thus should be treated.^101^ In this chapter, we critically review which and how nutritional approaches could ameliorate the course of IBD. Notably, diverse nutritional approaches make studies difficult to compare, and nutritional trials suffer from inadequate power with risk for bias, as summarised in a 2019 Cochrane review.^105^ Therefore, interpretation of many dietary intervention studies (and comparisons between them) must be made with caution, as discussed below.

**Table 1:** Characteristics of key nutritional trials in IBD

Inclusion of disease entity	Number of patients	Dietary intervention	Groups	Duration	Results of the main end-point(s)	References
*Exclusive enteral nutrition (EEN)*						
Paediatric CD cases (age 3–17 years) with weighted Paediatric CD Activity Index score (wPCDAI) >40	100 paediatric CD	Group1 (FL-IFX): Five infusions of 5 mg/kg IFX. Group2 (Conventional): EEN or Oral prednisolone (1 mg/kg, maximum 40 mg)	Group1: 50 Group2: 50	52 weeks	FL-IFX was superior to conventional treatment in achieving short-term clinical and endoscopic remission, and had greater likelihood of maintaining clinical remission	Jongsma *et al* 113
Children with new diagnosis CD	26 paediatric CD	EEN	**–**	6 weeks	EEN is effective for inducing early clinical, biochemical, mucosal and transmural remission. Early endoscopic remission improves outcomes at 1 year.	Grover *et al* 114
New-onset active CD (aged 6–17 years) with Harvey-Bradshaw Index (HBI) >5	19 paediatric CD	Group1: CS Group2: EEN	Group1: 6 Group2: 13	8 weeks	Both steroid and EEN induced clinical remission. Patients with EEN-induced remission showed a higher rate of mucosal healing and this was associated with a different gut microbiota compositional shift in these children.	Pigneur *et al* 115
Paediatric CD cases with a paediatric Crohn’s Disease Activity Index (PCDAI) >20	50 paediatric CD	Group1: 50% PEN with unrestricted diet. Group2: 100% TEN	Group1: 26 Group2: 24	6 weeks	TEN suppresses inflammation in active Crohn’s disease but PEN does not.	Johnson *et al* 127
*The Crohn’s disease and ulcerative colitis exclusion diet (CDED/UCED)*						
Paediatric CD cases with active disease (Paediatric Crohn’s Disease Activity Index >7.5 or Harvey-Bradshaw Index ≥4)	37 paediatric CD	CDED	–	6 weeks	Dietary therapy involving PEN with an exclusion diet lead to high remission rates in early mild-to-moderate luminal Crohn’s disease in children and young adults.	Sigall-Boneh *et al* 128
Children with mild to moderate CD	72 paediatric CD	Group1: CDED plus 50% of calories from formula for 6 weeks followed by CDED with 25% PEN for another 6 weeks. Group2: EEN for 6 weeks followed by a free diet with 25% PEN for another 6 weeks	Group1: 40 Group2: 38	12 weeks	CDED plus PEN was better tolerated than EEN in children with mild to moderate CD. The combination CDED plus PEN induced sustained remission in a significantly higher proportion of patients than EEN, and produced changes in the faecal microbiome associated with remission.	Levine *et al* 41
Adult patients with CD (aged 18–55 years) with mild-to moderate CD (defined by a Harvey-Bradshaw Index score of 5–14 points)	44 adult CD	Group1: CDED plus PEN Group2: CDED alone	Group1: 20 Group2: 24	24 weeks	68% of patients treated with CDED plus partial enteral nutrition achieved clinical remission, which was also achieved in 57% of patients with CDED alone.	Yanai *et al* 39
Adult patients with active UC (Simple Clinical Colitis Activity Index (SCCAI) of ≥5 and ≤11 and endoscopic Mayo score 2–3)	51 adult UC	Group1: Free diet plus FT. Group2: FT with dietary pre-conditionning of the donor for 14 days and a UCED. Group3: UCED alone	Group1: 17 Group2: 19 Group3: 15	8 weeks	UCED alone appeared to achieve higher clinical remission and mucosal healing than single donor FT with or without diet.	Sarbagili Shabat *et al* 43
Children diagnosed with CD	61 paediatric CD	Group1: CDED plus PEN (80% with prior 1–2 weeks of EEN) Group2: EEN	Group1: 20 Group2: 41	6–8 weeks	Treatment with CDED+PEN (with prior 1–2 weeks of EEN) has comparable efficacy to EEN therapy alone in inducing remission in children with CD, and it leads to better weight gain.	Niseteo *et al* 131
Patients with CD with loss of response (LoR) to biologics	21 CD (11 adults and 10 children)	Partial enteral nutrition (PEN)+CDED (severe paediatric patients recieved prior 14 days of EEN)	–	12 weeks	Dietary treatment combining PEN with the CDED may be a useful salvage regimen for patients failing biological therapy despite dose escalation.	Sigall Boneh *et al* 133
*The specific carbohydrate diet (SCD)*						
Paediatric patients (aged 10–17 years) with mild to moderate IBD defined by Paediatric Crohn’s Disease Activity Index (PCDAI 10–45) or Paediatric Ulcerative Colitis Activity Index (PUCAI 10–65)	12 paediatric IBD	SCD	–	12 weeks	SCD therapy in IBD is associated with clinical and laboratory improvements as well as concomitant changes in the faecal microbiome.	Suskind *et al* 136
*The Mediterranean diet (MD)*						
Patients with IBD in remission	58 CD and 84 UC	MD	–	6 months	A reduction of malnutrition-related parameters and liver steatosis in patients with IBD after MD, which associated with a spontaneous improvement of disease activity and inflammatory markers.	Chicco *et al* 138
Adult patients with CD with mild-to-moderate symptoms	197 adult CD	Group1: SCD Group2: MD	Group1: 101 Group2: 96	12 weeks	The SCD was not superior to the MD to achieve symptomatic remission, FC response, and CRP response. Given these results, the greater ease of following the MD and other health benefits associated with the MD, the MD may be preferred to the SCD for most patients with CD with mild to moderate symptoms.	Lewis *et al* 42
*The low FODMAP diet (LFD)*						
IBD in remission or with mild-to-moderate disease and coexisting IBS-like symptoms (Rome III)	28 CD and 61 UC	Group1: LFD Group2: ND	Group1: 44 Group2: 45	6 weeks	A low-FODMAP diet reduced IBS-like symptoms and increased quality of life in patients with IBD in remission.	Pedersen *et al* 140
IBD in remission or with mild disease activity	35 CD and 20 UC	Group1: LFD Group2: SD	Group1: 26 Group2: 29	6 weeks	LFD is safe for patients with IBD, and is associated with an amelioration of faecal inflammatory markers and quality of life	Bodini *et al* 141
*The gluten-free diet (GFD)*						
Patients with IBD	106 patients with IBD	Group1: GFD Group2: VD	Group1: 54 Group2: 52	–	No relevant impact of a specific diet on the course of the disease, but a significant association with lower psychological well-being in patients with VD and GFD.	Schreiner *et al* 144

This table summarises key aspects of recent nutritional trials in IBD.

CD, Crohn’s disease; CS, corticosteroid; FC, fecal calprotectin; FL, first-line treatment; FT, faecal transplantation; IBD, inflammatory bowel disease; IBS, irritable bowel syndrome; IFX, infliximab; ND, normal diet; PEN, partial enteral nutrition; SD, standard diet; TEN, total enteral nutrition; UC, ulcerative colitis; VD, vegetarian.

### Exclusive enteral nutrition

EEN takes advantage of an elemental (liquid) diet that meets all nutritional demands of macronutrients and micronutrients and thus allows replacing (solid) dietary habits. There are plenty of formulas available, which greatly vary in their composition of macronutrients and micronutrients.^106^ These formulas usually provide protein derived from whey and casein, simple carbohydrates from sucrose, maltodextrin or glucose syrup and fat from sunflower, soybean or fish oil, and they contain a range of food additives. In contrast, all formulas lack lactose and gluten and most of them lack fibre (complex carbohydrates). These formulas most significantly reduce energy intake derived from long-chain (saturated) fatty acids (when compared with dietary habits in the UK),^106^ which likely confers some of its efficacy.^107^ In this context, it appears notable that formulas contain a variable degree of monounsaturated fatty acid and PUFA enrichment.^106^

EEN is the recommended first-line therapy in children and adolescents with active (luminal) mild-to-moderate CD that is usually used for 6–8 weeks,^108–110^ with arguably comparable efficacy compared with corticosteroids.^111 112^ Efficacy between formula diets in mild-to-moderate CD appears comparable,^106^ while head-to-head trials are lacking. In contrast, there appears to be little therapeutic value in paediatric patients with severe CD.^113^ Notably, EEN can induce mucosal healing in mild-to-moderate CD (probably in ~50% of responders),^114^ which reflects a primary goal in medical trials.^115^ Monotherapy with maintenance enteral nutrition (ie, at least 50% of daily energy is derived from the formula diet) can prolong remission in paediatric CD.^109^ Indeed, mild small intestinal disease has the strongest predictive value of therapeutic response.^116^ EEN comprehensively impairs gut microbial diversity but increased its functional capacity,^117^ which appeared reversible after a switch to a standard diet.^118^ Notably, the microbiome and metabolome of responders to EEN differs from that of non-responders, suggesting the existence of a bacterial metabolic signature in some patients with CD.^119 120^ As such, the mode of action of EEN could involve anti-inflammatory functions of the gut microbiota, a mere reduction of (dietary or microbial) antigen load or a reduction of nutrient-induced immune responses.^121^ In contrast to a plethora of paediatric CD studies, little is known about the therapeutic efficacy of EEN in patients with UC.^122 123^

In adult patients with mild-to-moderate active CD, only few small studies suggest efficacy of EEN,^124^ which may nevertheless be recommended as an alternative to corticosteroids.^6 101^ For example, a randomised enteral nutrition trial in 55 adult patients with CD from Germany (published in 1991), demonstrated that an oligopeptide diet via nasogastric tube effectively induced clinical remission in 55% of patients in more and less severe disease (stratification by Crohn’s Disease Activity Index >300) after 6 weeks.^125^ This was however, less effective than corticosteroid and sulfasalazine therapy which induced clinical remission in 78% of patients with CD.^125^ Reduced efficacy of formula feed in adult IBD could generally be explained by impaired compliance (study discontinuation: ~40%) due to poor palatability or a distinct disease biology when compared with paediatric patients.^126^ Generally, EEN may induce mucosal healing and a clinical response in adult CD, which however could be confounded by co-medication, compliance issues and the lack of a placebo control (or study blinding).^124^ As such, these studies suggest that EEN improves gut inflammation in some adults with CD, the quality of evidence arguing for EEN is poor and prone to bias, which is why the routine use in adults is debated. This is also reflected by the fact that EEN in adult IBD is poorly depicted in current international consensus guidelines.

### The Crohn’s disease and ulcerative colitis exclusion diet (CDED/UCED)

A study in paediatric CD published in 2006 indicated that unrestricted partial enteral nutrition in combination with an elemental formula was less effective in inducing remission than EEN.^127^ Thus, it was conceived that a specific exclusion diet, which reduces or eliminates potentially detrimental food items (based on experimental evidence), would allow partial enteral nutrition that increases compliance with long-term dietary advice.^128 129^ Indeed, this concept is superior in Israeli and Canadian children with CD when compared with EEN.^41^ In this prospective study with 78 mild-to-moderate paediatric patients with CD, an elemental formula provided 50% of calories, while dietary advice with restriction of Western food items (to reduce an excess of animal fat, deep fried and processed food, dairy, emulsifiers, artificial sweeteners, soft drinks and wheat) provided the rest of calories in the first 6 weeks. This CDED then served as dietary maintenance therapy (with 25% of calories from an elemental formula) for another 6 weeks. CDED with partial enteral nutrition (with an elemental formula) was better tolerated and more effective after 12 weeks when compared with EEN for 6 weeks (followed by a free diet with 25% of calories from an elemental formula).^41^ More specifically, ~75% of patients receiving CDED with partial enteral nutrition were in steroid-free clinical remission, which was associated with microbial alterations such as reduced abundance of Proteobacteria. This approach was further tested in a prospective study comprising 44 adult patients with mild-to-moderate CD who were allocated to receive either CDED plus an elemental formula or CDED alone for 24 weeks. This study demonstrated after 6 weeks that 68% of patients treated with CDED and an elemental formula achieved clinical remission, which was also achieved in 57% of patients with CDED alone. Notably, clinical remission was maintained up to 24 weeks in 80% of the ‘responders’, and 35% of patients with CD achieved endoscopic remission at that time.^130^ These studies indicate that CDED may be recommended in paediatric CD (in combination with EEN), while evidence in adult IBD is less conclusive. Notably, these dietary intervention studies investigated their use for induction of remission, but not long-term efficacy. This is notable because such restrictive diets are considered to promote poor or disordered eating behaviour and possibly malnutrition, complications that may be overlooked in the reported short-term studies. These caveats underline the importance of dietary guidance by specialised dietitians to avoid harm.^121^ Notably, it is also unclear whether these diets are helpful for maintenance of remission.

In active mild-to-moderate UC patients that were refractory to therapy (ie, aminosalicylates, corticosteroids, azathioprine or anti-tumour necrosis factor antibodies), a blinded, randomised, controlled trial with 62 participants from Israel and Italy investigated whether an exclusion diet with or without faecal microbiota transplantation could be effective. The UCED required dietary counselling that recommended enrichment of fruits and vegetables and disallowed Western dietary habits (eg, intake of processed or ready-made food and twice a weak chicken breast or fish). The study was terminated early because the primary hypothesis that such a dietary approach would be beneficial in combination with faecal microbiota transplantation was rejected. However, the restriction diet alone induced clinical remission in 40% and endoscopic remission in 26% of patients at week 8 in this therapy refractory cohort,^43^ providing a basis for future nutritional studies in UC.

Collectively, these early clinical trials provide evidence that the diet impacts gut inflammation in mild-to-moderate CD and UC, and real-world experience suggested that dietary approaches are efficacious beyond clinical trials.^131^ The strengths of dietary therapy would be the easy access across the world, the low cost (probably 10%–30% compared with biologics in the first year) and, most importantly, avoidance of immunosuppression. Nutritional trials are also informative as they potentially allow to identify culprits of gut inflammation in IBD, as exemplified by reintroduction of meat and cereals which was associated with increased faecal calprotectin concentration after EEN in paediatric CD.^132^ Moreover, dietary approaches bear the potential to treat patients in whom biological therapies fail.^133^ Despite these observations, conclusive large clinical trials that would corroborate these concepts to establish evidence for an efficacious IBD diet are lacking. This approach is of utmost importance because published clinical trials are statistically underpowered (due to small cohort sizes), and they often lack a relevant comparator (eg, dietary counselling according to national guidelines). Moreover, current studies can neither depict nor delineate the heterogeneous response towards a restrictive diet in patients with IBD. As such early nutritional trials did not identify or resolve individual differences or disease phenotypes, which is required to approach the era of personalised nutrition. This may be partly explained by the lack of resources that are needed to execute studies that can compare with sponsored medical trials. Current studies also did not address whether more severe or complicated disease phenotypes would benefit from nutritional therapy (beyond correction of malnutrition), and whether a combination with medical therapy is beneficial. Finally, adherence to nutritional counselling must be evaluated to control for the bias of non-compliance, which can be frequently observed in daily practice and clinical trials alike.^134^ Overcoming these limitations will lead to evidence-based targeted nutritional therapies in IBD.

### The specific carbohydrate diet (SCD)

SCD is a restrictive grain-free diet which claims to maintain remission in patients with IBD. The diet allows digestible monosaccharide carbohydrates, which are made of a single molecule and easily to be broken down without enzyme participation, for instance contained in fruits, nuts, eggs, most (non-starchy) vegetables, non-processed meat and fish, while complex carbohydrates derived from grains, corn, milk and cream and artificial sweeteners are restricted. In a survey of 50 quiescent IBD subjects who employed an SCD for 10 months, complete symptom resolution by self-report appeared to be 66%.^135^ A study conducted with 12 paediatric patients with mild to moderate CD or UC subjected to an SCD diet demonstrated clinical improvement after 12 weeks, while two patients were unresponsive and two discontinued due to poor diet adherence. A distinctive dysbiosis for each individual in most pre-diet microbiomes ending in significant changes in microbiota composition after dietary switch. However, changes were not consistent in all patients.^136^ Besides these inconclusive studies, it was also hypothesised that an SCD could be efficacious from an observational point of view, since industrialisation of food production was paralleled by increased risk for IBD.^137^ Patients with IBD were wondering how an SCD compared with an Mediterranean diet (MD), which led investigators to initiate the DINE-CD study.^42^ In this North American study, 194 adult patients with CD with mild-to-moderate disease activity were randomised 1:1 to an SCD or MD and disease activity was evaluated after 6 and 12 weeks by clinical and biochemical (but not endoscopic) means. Self-reported adherence to either diet was ~65% and symptomatic and biochemical improvement (ie, faecal calprotectin <250 µg/g or reduction >50% from baseline) was observed in ~40% and ~30%, respectively, with both dietary regimen.^42^ However, C-reactive protein response was uncommon with both treatments. As such, further research is needed to understand which patient with IBD would benefit from an SCD, whether this diet affects harder endpoints (eg, endoscopic remission), and whether the reported response is sustainable. Notably, part of the effects of the diet may not be related to carbohydrates but correction of other Western dietary habits (eg, restriction of processed and canned or smoked meats and restriction of food additives). Based on the available evidence today, an SCD should not be recommended for patients with IBD.

### The Mediterranean diet

The MD is rich in arguably healthy foods including vegetables, fruits, legumes, cereals, fish and unsaturated fats. Results from clinical and translational research on the MD point towards a use in managing IBD.^100^ In a prospective Italian study comprising 84 patients with UC and 58 patients with CD in remission, all participants were counselled to adhere to an MD and disease was evaluated after 6 months by clinical and biochemical means. Quality of life improved for patients with CD and UC after 6 months, and patients appeared to have a reduced risk for a disease flare (concomitant to conventional medical therapy).^138^ In the DINE-CD study, 40% of patients with mild-to-moderate adult CD demonstrated clinical remission after 6 and 12 weeks (with little impact on biochemical inflammatory parameters), suggesting that an MD could be effective in some patients.^42^ Close adherence to an MD is associated with high level of beneficial *Prevotella* and fibre-degrading *Firmicutes*.^139^ The MD may be recommended for patients with IBD in remission, partly because of lack of evidence-based alternatives^101^ and a well-documented effect on cardiovascular disease, non-alcoholic fatty liver disease and depression.^121^ More clinical evidence should corroborate efficacy, safety and adherence in comparison to more stringent exclusion diets during active disease and remission to express this dietary recommendation with confidence.

### The low FODMAP diet (LFD)

Fermentable oligosaccharides, disaccharides, monosaccharides and polyols (FODMAPs) are short-chain carbohydrates contained in wheat, onion, cabbage, legumes and stone fruits that are poorly absorbed in the small intestine. A diet low in these fermentable carbohydrates is called an LFD. In 89 adult patients with IBD (28 CD, 61 UC) in remission or with mild-to-moderate disease, a randomised low FODMAP trial (vs a standard diet) for 6 weeks resulted in significant improvement in terms of quality of life and reduction of symptoms of concomitant irritable bowel syndrome.^140^ A similar prospective study with 55 IBD subjects (35 CD, 20 UC) demonstrated that an LFD reduced clinical disease activity in patients with mild disease (or in remission) when compared with a standard diet after 6 weeks.^141^ In a study with 9 patients with CD in remission, an LFD affected gastrointestinal symptoms and increased relative abundance of butyrate-producing *Clostridium* cluster XIVa and mucus-associated *Akkermansia muciniphila*.^142^ An LFD is currently recommended for patients with irritable bowel syndrome, but not for active IBD. Further clinical trials are needed to establish a clinical efficacy of an LFD to control gut inflammation in IBD.

### The gluten-free diet (GFD)

A GFD excludes all food items containing gluten, which is contained in wheat (and derivatives), barley, rye, triticale and brewer’s yeast, so that pasta, baked goods and beer (with other nuances) must be excluded from the diet. A cross-sectional questionnaire study with 1647 patients with IBD (with 0.6% concomitant coeliac disease or gluten-sensitivity) indicated that 20% have tried a GFD and that 66% of patients reported clinical improvement and 38% reported less flares.^143^ In contrast, a large prospective study involving 1254 patients with IBD in Switzerland reported no significant differences between patients who followed a GFD and those who did not, with regards to disease activity, complications, hospitalisation and surgery rates.^144^ A GFD is not recommended for patients with IBD.

## Conclusion and future directions

**Figure 2:**
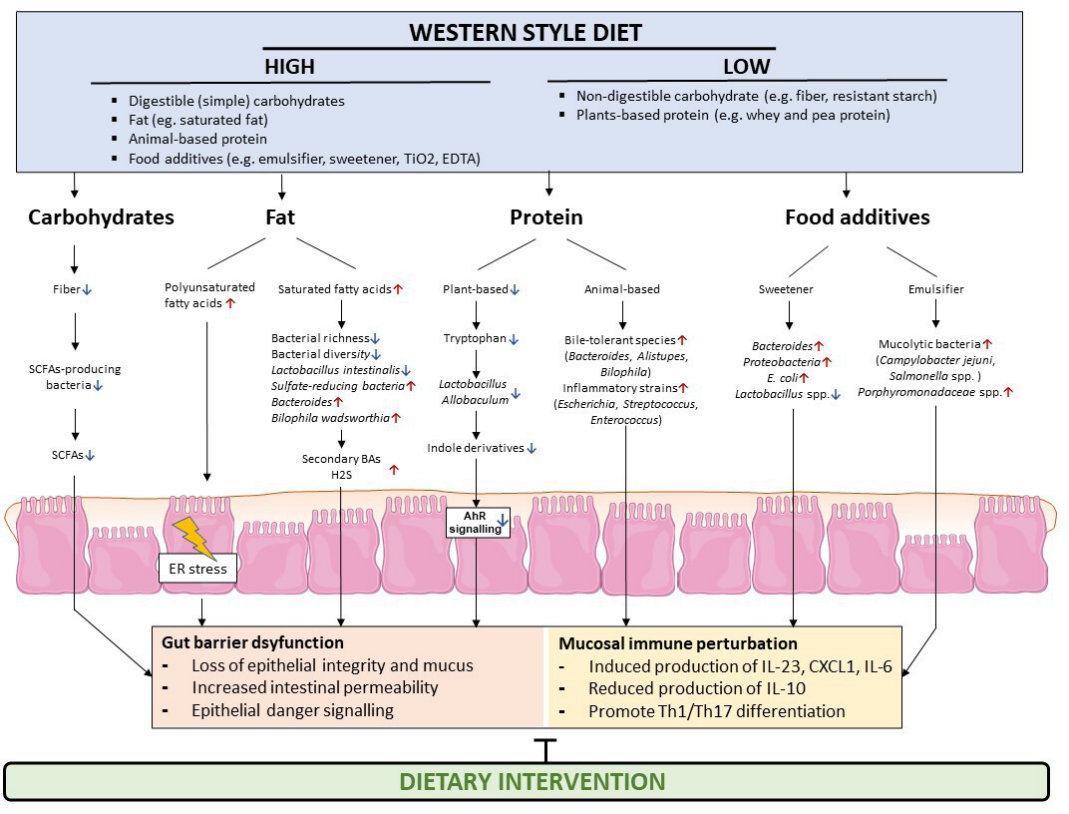
The diet and gut microbiota perturb immune responses in IBD. Dietary constituents such as macronutrients and food additives have been shown to affect the gut microbiota in humans. Diet-induced alterations of the gut microbiota may exert diverse effects on gut mucosal immune responses and IBD-associated dysbiosis promotes gut inflammation in preclinical models, partly by loss of production of beneficial microbial metabolites, such as SCFAs and indole derivatives. In addition, the bloom of certain pathobionts may impair the epithelial barrier and stimulate a proinflammatory environment. AhR, arylhydrocarbon receptor; BA, bile acid; ER, endoplasmic reticulum; H2S, hydrogen sulfide; IBD, inflammatory bowel diseases; IL, interleukin; SCFA, short chain fatty acid.

Preclinical and clinical studies from the last years demonstrated that the diet is a rheostat of microbial composition and function and may evoke dysbiosis, as exemplified by a human Western diet.^145^ Prime examples demonstrated that a specific dietary constituent triggers or deteriorates experimental gut inflammation in the context of genetic susceptibility, which is partly explained by gut microbial dysbiosis.^21 30 31 38^ Likewise, a dysbiotic microbiota from patients with IBD is fuelling an inflammatory response in the gut of mice.^34^ These studies collectively indicate that the diet and IBD-associated gut dysbiosis are tightly interrelated and control mucosal homeostasis by complex and context-specific immunomodulation through specific dietary constituents, microbial antigens or metabolites. In such a concept, heterogeneity of human IBD is not only related to genetic variation but also to a variable exposome (eg, the diet and gut microbiota) (figure 2). Nutritional trials in mild-to-moderate paediatric CD indicate that the diet is fuelling gut inflammation, because EEN with or without an exclusion diet (restricting Western dietary habits) effectively induces remission and allows mucosal healing in a substantial proportion of paediatric patients with CD, which arguably exhibits a comparable efficacy as immunosuppressive therapy. However, carefully designed nutritional studies of reasonable size, comparable to medical trials, are needed to disentangle disease heterogeneity and efficacy of nutritional therapy in adults with IBD, to overcome the limitations of dietary intervention studies of today. We propose a concept how to improve EEN formulas and the exclusion diet in CD and UC, which is largely based on preclinical evidence (tables 2 and 3). For example, elemental diets contain a range of food additives and they are largely deprived from fibre, both of which is known to compromise the gut microbiota and gut health.^106 146^ Moreover, elemental diets provide simple carbohydrates from sucrose and fat from fish oil, which demonstrated detrimental effects in mouse models of gut inflammation.^25 36 38 106^ These observations indicate the potential of basic research as a guide for novel nutritional concepts, which should be considered in the design of EEN formulas and future nutritional trials. While animal models imperfectly depict the complexity of our diet for gut health, they allow to study host–microbe interactions and related immune responses. Although difficult to translate, this approach will be rewarding, as only mechanistic insights in mammals allows to disentangle complex host–microbe interactions (shaped by the diet) that deserve to be exploited in controlled nutritional trials. Understanding the intricate interplay between the host and its commensals, and delineating the impact of specific dietary factors on this interplay, will also set the ground for our phenotypic understanding of heterogenous IBDs and at the same time bears the potential to prevent IBD as it would allow informed decision making in food politics. And refinement and corroboration of existing dietary therapy in IBD harbours the potential to avoid immunosuppressive treatment (with related side effects and costs). When compared with coeliac disease, it appears unlikely that one diet suits most patients, which is another reason to perform large scale nutritional trials to specifically define disease phenotypes (or traits) that are responsive to nutritional therapy. Thus, future nutritional trials should not only evaluate long-term efficacy, safety and dietary adherence to overcome limitations of EEN (eg, poor palatability), but also establish quantitative and reproducible tools beyond dietary questionnaires to allow monitoring of food intake that are not prone to recall bias, such as blood and stool metabolomics. Advances in this field will change the perception of IBD, and will allow identification of nutritional phenotypes, which may enable us to enter the era of personalised nutrition. To achieve this, scientists and practitioners should not only revisit their perception of the diet in IBD, but stake holders should take action. This step appears critical because nutritional trials should scientifically hold up with medical trials in IBD, which requires dedication from nutritional sponsors and support from policy makers. That this may be rewarding for individuals, and socio-economically, has been recognised by other fields and will change nutritional practice, as for example in oncology.^147^ The concept of precision nutrition is expected to change the way we understand and treat IBD.

**Table 2:** Proposed common ground for dietary therapy in CD and UC

	Rationale	Recommendation
*Disallow*		
Artificial sweetener (sacchrine, splenda)	Experimentally promoting gut inflammation,^92 94^ altering human gut microbiota,^93^ restriction in nutritional trials^39–41^	Stop ultra-processed, ready-made or canned food, sweets, soft drinks
Emulsifiers (P80, CMC)	Experimentally promoting gut inflammation,^31^ altering human gut microbiota,^96^ restriction in nutritional trials^39–41^	Stop ultra-processed, ready-made or canned food, sweets, soft drinks
Food colourants (Red 40/E129, Yellow 6/E110)	Experimentally promoting gut inflammation,^30^ restriction in nutritional trials^39–41^	Stop ultra-processed, ready-made or canned food, sweets, soft drinks
Ultra-processed food	Experimentally promoting gut inflammation (see additives above), restriction in nutritional trials^39–41^	Stop ultra-processed, ready-made or canned food, sweets, soft drinks
*Restrict*		
Saturated and polyunsaturated fatty acids	Experimentally promoting gut inflammation,^36 38^ arguably restriction in nutritional trials^39–41^	Restrict animal fat (regardless of source), deep fried and ultra-processed food
Sucrose, Glucose, Fructose	Experimentally promoting gut inflammation,^23–27^ arguably restriction in nutritional trials^39–41^	Restrict soft drinks, sweets, ready- made food
*Enrich*		
Plant-based food items (fibre source)	Enrichment in nutritional trials^39–41^	Encourage plant-based diet

Dietary counselling recommendation based on experimental evidence and nutritional trials. Note that the efficacy and safety of the proposed dietary alterations requires corroboration by controlled nutritional trials in patients with IBD.

CD, Crohn’s disease; IBD, inflammatory bowel disease; UC, ulcerative colitis.

**Table 3:** Potential inflammatory nutrients in elemental diets

	Rationale
*Remove or reduce*	
Milk fat	Experimentally promoting gut inflammation^21^
Fish oil	Experimentally promoting gut inflammation^36 38^
Soybean oil	Experimentally promoting gut inflammation^36 38^
High omega-3 or omega-6 PUFA oil	Experimentally promoting gut inflammation^36 38^
Maltodextrin	Experimentally promoting gut inflammation^32^
*Enrich*	
Fibre (eg, inulin and fructooligosacharides)	Experimental and clinical evidence^87^
Plant-based protein	Experimental evidence,^89^ human gut microbiota modulation^87^
Olive oil	Experimental evidence^153 154^

Elemental diets (formula feed) contain potentially inflammatory nutrients indicated by experimental studies. These studies suggest to restrict or enrich specific food constituents to improve efficacy. Note that an elemental diet should be used in conjunction with counselling by nutritionists and that the efficacy and safety of the proposed regimens require corroboration by controlled nutritional trials in patients with IBD.

IBD, inflammatory bowel disease; PUFA, polyunsaturated fatty acid.
